# A community based prevention of weight gain intervention (*Mothers In Motion*) among young low-income overweight and obese mothers: design and rationale

**DOI:** 10.1186/1471-2458-14-280

**Published:** 2014-03-25

**Authors:** Mei-Wei Chang, Susan Nitzke, Roger Brown, Ken Resnicow

**Affiliations:** 1 Michigan State University, College of Nursing, 1355 Bogue Street, RM C346, East Lansing, MI 48824, USA; 2 Department of Nutritional Sciences, University of Wisconsin-Madison, 1415 Linden Drive, Madison, WI 53706, USA; 3 University of Wisconsin-Madison, School of Nursing, 600 Highland Avenue, Madison, WI 53792, USA; 4 University of Michigan, School of Public Health, 1415 Washington Heights, Ann Arbor, Michigan 48104, USA

**Keywords:** Obesity prevention, Stress management, Healthy eating, Physical activity, Low-income women

## Abstract

**Background:**

Over 45% of American women 20–39 years old are at risk for type 2 diabetes, cardiovascular disease, and other health conditions because they are overweight or obese. The prevalence of overweight and obesity is disproportionately high among low-income women. This paper describes the study design and rationale of a community based intervention (*Mothers In Motion, MIM*) aimed to prevent weight gain among low-income overweight and obese mothers18-39 years old by promoting stress management, healthy eating, and physical activity.

**Methods/Design:**

Peer recruiters approach participants from 5 Special Supplemental Nutrition Program for Women, Infants, and Children (WIC) in Michigan. The *MIM* delivers theory-based, culturally-sensitive intervention messages via a combination of DVDs and peer support group teleconferences (PSGTs). The DVD features African American and white overweight and obese WIC mothers who participated in a healthy lifestyle intervention patterned after *MIM*. The PSGTs are led by paraprofessionals from Michigan State University Extension and WIC providers in Michigan who are trained in motivational interviewing and group facilitation skills. Participants are randomly assigned to an intervention (n = 350) or comparison group (n = 175). The intervention group receives a 16-week intervention on a weekly or bi-weekly basis. Participants are asked to watch 10 *MIM* DVD chapters at home and join 10 PSGT sessions by phone. The comparison group receives printed educational materials. The primary outcome is body weight. Secondary outcomes include dietary fat, fruit, and vegetable intake; physical activity; stress, and affect. Mediators are self-efficacy, emotional coping response, social support, and autonomous motivation. Telephone interviews and in-person data collection at WIC offices occur at 3 time points: baseline, immediately, and 3 months after the 16-week intervention.

**Discussion:**

If *MIM* shows effectiveness, it could have a favorable impact on public health and community programs. The DVDs and PSGTs will be disseminated in WIC, Extension, clinical practice that promote healthy lifestyles for similar target audiences to make a broad contribution to the prevention of weight gain in low-income mothers. Also, our methodology can be adapted by researchers and community stakeholders to help other low-income populations prevent weight gain.

**Trial registration:**

Clinical Trials Number: NCT01839708.

## Background

Over 45% of American women 20–39 years old are overweight or obese placing them at risk for several chronic diseases, e.g., type 2 diabetes mellitus (DM) and cardiovascular disease (CVD) [1-4]. The postpartum period is a critical time when weight retention and weight gain can lead to long-term increases in adiposity [5,6], exacerbating health problems related to overweight and obesity. Most low-income mothers (85%) retain 15 lbs at 6 weeks postpartum [7,8]. On average, women retain at least 2.2-4.4 lbs after 6–18 months postpartum or beyond [9,10]. Also, low-income overweight and obese women are at risk of major weight gain (> 25 lbs) 10 years after delivery [11,12]. Unfortunately, current weight loss programs remain relatively ineffective for treating obesity, as participants ultimately regain their lost weight [13,14]. Also, few programs that have targeted the needs of low-income mothers have been tested [15]. Thus, preventing weight gain in women with a high risk of major weight gain [10] is a high priority [3,16]. Excess weight gain in early adulthood (aged 20–30) increases CVD risk factors [17], whereas preventing weight gain is associated with either improving or not significantly changing the risk of developing CVD [3,18], and is generally easier to achieve than weight loss [3]. Also, preventing an increase of even 1 body mass index (BMI) unit (~6 lbs) can prevent a 13% increased risk for developing type 2 DM [1]. Moreover, preventing weight gain has societal benefits by reducing the cost burden of DM treatment and losses in productivity [19].

The Diabetes Prevention Program (DPP) demonstrated that healthy lifestyle (healthy eating and physical activity) promoted weight loss (~7%) [20]. However, the DPP’s intervention model, which entailed 16 intensive counseling sessions is not readily implemented in typical social service settings where providers face competing demands as they care for a diverse audience with limited resources [21]. Additional barriers to translating evidence to practice include lack of cultural competence and underutilization of information technology [22]. We developed the *Mothers In Motion (MIM)* with the potential for implementation in community based settings, e.g., the Special Supplemental Nutrition Program for Women, Infants, and Children (WIC). WIC is a federally funded program that provides nutrition consultation and other services to low-income pregnant, postpartum, and breastfeeding women and children less than 5 years.

To address cultural misunderstandings, we include deep and surface structure components of low-income African American and white culture in the *MIM* DVDs*.* Deep structure reflects how cultural, social, psychological, historical, and environmental factors influence health behaviors differently across racial populations [23,24]. The deep structure requires understanding how the target audience perceives the cause, course, and treatment of illnesses (e.g., overweight and obesity) and how their perceptions influence specific healthy lifestyle behaviors (e.g., not being physically active because of time constraints) [25]. The surface structure includes incorporating an appropriate channel of intervention and using people, language, foods, clothing, music, and locations that are familiar to and preferred by the target audience.

To overcome underutilization of information technology, we use DVD and peer support group teleconference (PSGT) to deliver the intervention. Researchers have used an interactive CD-ROM to deliver a single dose of nutrition education to WIC mothers in WIC offices [26-29] and found significant improvements in nutrition attitudes [26,27] and self-efficacy [28], but not dietary behavior change [28]. However, a study of diabetic patients using a combination of interactive CD-ROM and individual telephone consultation found significantly reduced dietary fat intake, increased fruit and vegetable intake, and increased physical activity [30]. These studies suggest that repeated contact and a combination of these modes might be needed. However, repeated contact with interactive CD-ROM in WIC offices is not feasible [28]. An alternative is to watch DVDs at participants’ homes, given the fact that most American households including low-income have a television (99%) [31]. Our pilot *MIM* showed that more than 90% of WIC mothers had a DVD player at home [32]. Most low-income people list TV as a major source of health information [33]. The DVD, which will not require conventional prose literacy skills [34], is an effective means to deliver motivational and educational messages to low-income populations and is cost effective [35].

Interventions utilizing PSGTs have resulted in significant gains in coping, support satisfaction, perceived support and information [36-38], and decreased feelings of isolation and loneliness [36,39]. The teleconference environment is comfortable and convenient, provides privacy, is readily accessible without transportation, and can increase participation [40,41]. Also, group intervention can be effective because it provides empathy, social support, and a healthy dose of competition [13]. Thus, the combination of DVDs and PSGTs has great potential for promoting healthy lifestyle and meeting the needs of low-income mothers. This was shown in our pilot *MIM*[32]. This paper describes the study design and rationale of a community based intervention (*MIM*) aimed to prevent weight gain among low-income overweight and obese mothers 18–39 years old by promoting stress management, healthy eating, and physical activity.

### Hypotheses

Hypothesis 1: The intervention group will have lower weight gain (difference between the baseline and 3 months post intervention) than the comparison by an average of at least 2.8 lbs, due to a combination of prevention of weight gain in the intervention group and a slight weight gain in the comparison group.

Hypothesis 2: At 3 months post intervention, the intervention group will have greater improvements than comparison group members in dietary fat, fruit and vegetable intake; physical activity; stress; affect; self-efficacy; emotional coping response; social support; and autonomous motivation.

## Methods/Design

**Figure 1:**
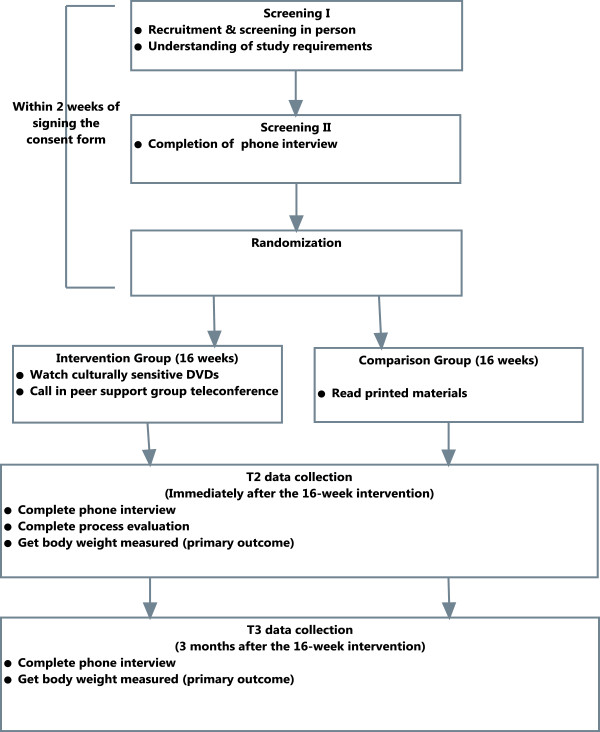
Mothers in motion study design.

Figure 1 presents study design of the *MIM*. The study (recruitment, intervention and data collection) is being conducted with the approval of 2 Institute Review Boards at Michigan State University and Michigan Department of Community Health. We applied community based participatory approach to design the intervention and study methods and continue to work closely with our community and peer advisory groups. The key function of these groups is to assist in planning and evaluating intervention and to review rough-cut DVDs. The community advisors also help with *MIM* recruitment, training of PSGT moderators, evaluation and implementation. The peer advisory group includes 6 African American and white overweight or obese WIC mothers. The community advisory group (N = 6) includes WIC administrators from the collaborating WIC agencies and the State of Michigan WIC.

### Setting, participants, and recruitment

Participants are recruited from 5 collaborating WIC regional agencies (comprising 9 WIC offices) in Michigan; 75.5% of WIC enrollees from these agencies received Medicaid. Women coming to these WIC agencies during the data collection dates are personally invited by peer recruiters to participate while waiting for appointments. The recruiters are trained in communication skills. They explain the study purpose (e.g., help participants have a happier and healthier family), confidentiality and study requirements and emphasize *MIM*’s flexible schedule and its easy, no-cost availability. Inclusion criteria. Participants must be non pregnant African Americans or non Hispanic whites, understand and speak English, be 18–39 years old, be at least 6 weeks postpartum, be willing to provide valid contact information for 3 back-up people, be willing to update their contact information monthly, be willing to participate in the *MIM* for 9 months, be willing to make 3 additional trips to the WIC offices where they were recruited for randomization and body weight measured, have a working DVD player or computer at home, and access to a working phone. Also, participants must have a measured body mass index (BMI) between 25.0 and 39.9 kg/m^2^. Height was measured to the nearest 0.1 cm using a wall-mounted stadiometer, with participants wearing no shoes. Weight was measured using an electronic digital scale (described later). Exclusion criteria. We exclude women who plan to become pregnant or relocate to a location outside of study sites during the trial, have a self-reported type 1 or 2 DM, or are unable to walk more than 1 block without resting or shortness of breath.

To minimize a potential dropout, we implemented a sequential screening based on lessons learned from the pilot *MIM*[32]: one of key reasons for high dropout was not fully understanding the study requirements and incentives. Screening I. We conducted cognitive testing with 20 WIC mothers to develop an easy-to-read flyer that provided a study overview. Eligible participants read the flyer, then are interviewed (~2 minutes) by the recruiter to assess their interest and commitment and to determine if the study goals and expectations are a good fit. The recruiters ask eligible participants to sign a consent form only if they could verbalize full understanding of the study requirements. Screening II. Eligible participants are enrolled after they complete baseline measures and return to the WIC offices where they are recruited in person to pick up the study package within 2 weeks after signing the consent form.

### Randomization

Within 2 weeks of signing the consent form, participants who complete baseline measures (described later) return to their WIC offices to be randomized to either the intervention or comparison group. The peer recruiters use a laptop computer equipped with a WIFI to perform randomization. Then, participants receive either intervention or comparison study package and gift incentives (e.g., cooking utensil or detergents).

### Intervention

#### Culturally sensitive DVDs

The video portrays the experience of four WIC mothers (3 African Americans and 1 white, hereafter refer as featured mothers) who met the study criteria. They were counseled and encouraged for 16 weeks (~3-4 hours/per week) to make healthy lifestyle changes in 3 key areas of daily life: stress management, healthy eating, and physical activity. The first author applied motivational interviewing (MI) techniques and concepts from the Social Cognitive and Self-Determination Theories (e.g., self-efficacy, emotional coping response, and autonomous motivation) to conduct counseling. Each session included 1) setting a small, realistic, and measurable goals, 2) asking open-ended questions to make featured mothers think about their current lifestyle, identify root causes of their problems, and 3) developing problem solving skills. All counseling sessions and filming took place at the featured mothers’ homes, play grounds, neighborhoods and local grocery stores. We filmed the 4 featured mothers, their significant others, and young children over 1 year to document changes over time: before, during, 3 and 6 months after the 16-week counseling. Each filming included 2 parts: interview and B-role (action). The interview questions were developed based on the principle of MI and B-roles were based on the content of interview.

The final DVD set has 11 intervention chapters. Each topic was designed to be independent with no overlapping content. *Introductory chapter* (~6 minutes) presents the study purpose, a preview of the *MIM* DVDs, and goal setting that emphasizes importance, confidence, core values, and small/gradual healthy lifestyle change. *Ten intervention chapters* (~20 minutes/chapter) address stress management (n = 4), healthy eating (n = 5), and lifestyle physical activity (n = 1). Each chapter has 3 components with a similar structure: interactive information (~2 minutes), culturally-sensitive vignettes (~17 minutes), and action plan (~40 seconds). We also include a bonus component (1–2 minutes per chapter, e.g., stress and anger logs, recipes, or credible websites). The interactive information and action plan have young female African American and white voice over to reduce literacy barriers to learn. The stress management chapters include better ways to handle everyday responsibilities and hassles (e.g., laundry and dishes), time saving tips (e.g., setting priority), ways to handle negative feelings (e.g., emotional eating), and parenting tips. The healthy eating chapters cover effective ways to reduce junk food intake, read food labels, plan meals, shop for grocery, and cook healthier with children. The physical activity chapter includes fun and realistic ways to get more lifestyle physical activity (low to moderate) both at home and outdoors (e.g., marching while watching TV, and parking farther away from building).

The interactive information is designed to correct misperceptions, increase self-awareness of current lifestyle, and promote a healthy lifestyle. We use ‘pause’ and ‘continue’ buttons to allow viewers to stop and think about their current lifestyle. Culturally-sensitive vignettes are designed to engage the attention of the audience with poignant story lines, real world examples, and testimonials that model healthy lifestyle. The featured mothers demonstrate ways to identify root causes of their problems, positive and negative experiences in making changes and their own lifestyle affecting their family members. We include testimonials from the featured mothers, their significant others and young children (3–9 years). We also present real stories before, during, and after making changes and the benefits therein (e.g., impact on young children) over 1 year. Moreover, we show strategies to overcome procrastination and challenges of making change.

Each section concludes with an action plan, which is presented in a multiple choice format and is used to monitor participants’ compliance of watching each DVD chapter. The action plan focuses on key strategies related to self-efficacy from each chapter and helps viewers make action plans for healthy lifestyle changes. The first rough-cuts of DVDs were reviewed by community partners and overweight or obese WIC mothers (N > 50) and their feedback was used to revise the DVDs. The second rough-cuts were reviewed by 3 focus groups (5–6 African American and white overweight or obese WIC mothers/per group). Their feedback was used to finalize the DVDs and the featured mothers approved the DVD content.

#### Peer support group teleconference

The PSGT moderators attended a 3-day training addressing both MI and group facilitation skills via a mix of didactic and experiential/practice activities. About 2–3 weeks before leading the actual PSGTs, each moderator took part in 4 supervisory sessions by telephone. They led the discussions with WIC mothers who met the study criteria. MI trainers provided immediately feedback for improvement after each practice call. The trainers also listen to 25% randomly selected PSGT recordings/per month and provide feedback for improvement. The moderators attend booster training quarterly.

#### 16-week Intervention

Regardless of group assignment, each study participant receives usual WIC care which includes nutrition counseling for their young children. *Intervention group.* DVDs. Intervention participants watch 1 intervention chapter weekly (Weeks 1–4) or bi-weekly (weeks 5–16) at home, except the first week that they also watch the introductory chapter. After watching a DVD chapter, the participants answer 3 questions (action plan) and mail the action plan worksheet to the study office using a pre-stamped envelope so that we could monitor compliance of watching the intervention chapters. The participants also use a weekly worksheet to set 1–2 personal goal(s) and to make plans for behavioral changes and self-monitor progress for 7 days.

Based on the pilot *MIM*[32], we create groups comprised of about 10 women who remain in the same cohort for the 16-week intervention. The participants dial into the PSGT weekly or bi-weekly and discuss contents in the designated DVD chapter watched. To increase participation, they are provided 2 different times (one in mid morning or early afternoon and one in the evening) to call into PSGT. Each PSGT session lasts about 30 minutes. The moderators are on-line as participants call in. Their roles are to establish a safe, non-confrontational, and supportive climate where the participants feel comfortable discussing the stress in their lives and expressing both positive and negative aspects of their current behavior, and the pros and cons for change. They also help participants explore their ambivalence about change and motivate participants address their own barriers. They followed a semi-structured “roadmap protocol”, e.g., introduction, rapport building, assessment of current behaviors and progress and providing feedback with permission from participants. The assistant moderator records the session, takes notes, keeps time, and uses a log to keep track of who calls in to monitor PSGT compliance.

##### Comparison group

The comparison group receives printed materials from standard reliable sources such as MyPlate.gov for stress management, healthy eating, and physical activity. They also receive a food and home safety DVD (~10 minutes). We provide printed materials to the comparison group to avoid confusion and make both groups’ information more parallel in scope. During the recruitment, we tell the potential participants that the *MIM* program is about healthy eating, physical activity, and stress management for a happy and healthy family. We are concerned that the comparison participants may drop out if they do not receive educational materials that fit this description.

### Measurements

Data were collected via phone or in person (body weight measured at WIC offices) at 3 time points: baseline (T1), immediately (T2) and 3-month (T3) after the 16-week intervention. Primary outcome. Body weight is measured to the nearest 0.2 pounds on an electronic digital scale (Seca 869, Germany) with the participants wearing light clothing and no shoes. Secondary outcomes. We measure dietary fat, fruit and vegetable intake, physical activity, perceived stress, and affect. To measure dietary fat, fruit and vegetable intake, we use Rapid Food Screener (24 items: 17 items for dietary fat, 7 items for fruit and vegetable intake) which has adequate validity and reliability [42]. Each month, the first 7 women who complete the T1 phone interview also complete Block’s short form Food Frequency Questionnaire (FFQ) [43]. Physical activity is measured using Pregnancy Infection Nutrition (PIN) 7-day physical activity recall (asking frequency, duration, and intensity) with established validity and reliability [44]. Perceived stress is measured using a 9-item Perceived Stress Scale [45]. Affect is measured using the Positive Affect and Negative Affect Scale (18 items) [46]. Mediators. Mediators include self-efficacy, emotional coping response, social support and autonomous motivation. Self-efficacy is measured by asking participants’ to rate their confidence (1 = not confident at all, 4 = very confident) in eating healthy foods (8 items), becoming more physically active (10 items), and managing their stress (9 items) [47,48]. Emotional coping response is measured by asking participants about strategies used to cope with emotional eating (6 items), physical activity (4 items), and stress management (7 items) [47,49]. Social support is measured by asking participants to report social support from their family members, friends, or other people to eating healthy foods (6 items), engaging in physical activity (4 items), and managing stress (6 items) [47]. Autonomous motivation for healthy eating (17 items), physical activity (17 items) and stress management (17 items) are measured using Treatment Self-Regulation Questionnaire [50]. Process evaluation. We perform process evaluation (T2 only, e.g., program satisfaction, DVD watched at home, and reasons DVDs not watch or dial in PSGT) to help explain intervention impact.

### Sample size and statistical methods

The sample size calculation was based on a two-sided test of significance at α < 0.05, 80% power, a difference in mean change in body weight between groups of 2.8 pounds and standard deviation of weight change = 10.7 pounds [32], and an allocation rate of 2 (intervention):1 (comparison). Since we anticipated 30% attrition and 15% pregnancy [51] occurrence during the study, we will oversampled up to 525 women to reach 290 the final sample size. For data missing either at completely random or at random, we will use the Mplus modeling package to impute data by implementing the full information maximum likelihood algorithm. We will perform intention to treat analysis. Also, we will use appropriate statistical tests (e.g., t-test and analysis of covariance model for continuous variables and chi-squared test for categorical variables) to confirm the success of randomization. To assess 2 hypotheses at 3 months post intervention, a general linear model will be used covarying mean centered baseline measures, including a treatment condition dummy variable as well as covariates.

## Discussion

WIC has a strong interest in obesity prevention. Thus, this study's findings should be of interest to a broad audience of policy makers and could lead to a variety of dissemination initiatives. Based on the results of this study, the research team and WIC will conduct a series of activities aimed at sharing the findings and collaboratively interpreting and translating the findings into their usual practice and a set of policy recommendations. Assuming effectiveness, the research team will work with WIC to refine the *MIM* components. This may include refining elements and revising content of the *MIM* DVDs and the PSGT training manuals, and other materials as needed then turn over to WIC for implementation. Being aware that recruiting mothers into this type of program is a challenge, we will work with community and peer advisory groups to create a culturally-sensitive recruitment DVD and flyers/pamphlets and postcards/subscription system to encourage African American and white overweight or obese mothers to enroll in the *MIM*. To meet the ultimate translational goals, we will initially target WIC at state/district level. We will also consider targeting more clinically oriented programs that serve this audience (e.g., pediatric and OB/GYN offices) and to other settings (e.g., Community Health Centers, churches, and Head Start). We will offer trainings via interactive internet technology (webinars) to representatives from WIC and others at the state/district level. The webinars will be videotaped for those who cannot participate. We will use a “train the trainer” model to organize the training conference, thus representatives would complete training of the PSGT protocol and implementation of the *MIM* DVDs. They would then be charged to train the others at their state/district. Additionally, a set of recommendations for effective dissemination and implementation of the *MIM* will be distributed via print, Web, and other channels.

If *MIM* shows effectiveness, as we anticipate it will, it will have a favorable impact on public health and community programs. The DVDs and PSGTs will be disseminated in WIC, Extension, clinical practice (e.g., OB/GYN and pediatric offices), and other settings (e.g., Community Health Centers, churches, and Head Start) that promote healthy lifestyles for similar target audiences to make a broad contribution to the prevention of weight gain in low-income mothers. Also, the intervention’s methodology can be adapted by researchers and community stakeholders to help other low-income populations (e.g., Hispanic and Asian) prevent weight gain. We also anticipate a concomitant benefit on childhood obesity because promotion of mothers’ healthy lifestyles can help their young children establish healthy eating behaviors and physical activity, which are major factors in preventing childhood obesity.

## Abbreviations

BMI: Body mass index; CVD: Cardiovascular disease; DM: Diabetes mellitus; DPP: Diabetes prevention program; MI: Motivational interviewing; MIM: Mothers in motion; PSGT: Peer support group teleconference; TV: Television; WIC: The special supplemental nutrition program for women, infants, and children.

## Competing interests

The authors declare that they have no competing interests.

## Authors’ contributions

MC, SN, RB, and KR conceived and designed the study and all four submitted the proposal for funding. MC and KR substantially contributed to the DVD development. MC, SN, and RB substantially contributed to the operationalization of the study once funded. MC drafted and revised the manuscript and SN, RB, and KR involved revision. All the authors have read and approved the final manuscript.

## Pre-publication history

The pre-publication history for this paper can be accessed here:

http://www.biomedcentral.com/1471-2458/14/280/prepub
